# In Vitro Methods for Assessing the Antibacterial and Antibiofilm Properties of Essential Oils as Potential Root Canal Irrigants—A Simplified Description of the Technical Steps

**DOI:** 10.3390/mps7040050

**Published:** 2024-06-25

**Authors:** Jihad Diouchi, Jelena Marinković, Milica Nemoda, Lhoussaine El Rhaffari, Babacar Toure, Sonia Ghoul

**Affiliations:** 1 Health Sciences Research Center, International Faculty of Dental Medicine, College of Health Sciences, International University of Rabat, Technopolis Parc, Rocade of Rabat-Salé, Sala-Al Jadida 11100, Morocco; babacar.toure@uir.ac.ma (B.T.); sonia.ghoul@uir.ac.ma (S.G.); 2 National Institute of the Republic of Serbia, ‘VINCA’ Institute of Nuclear Sciences, University of Belgrade, Mike Petrovica Alasa 12, 11000 Belgrade, Serbia; jelena.marinkovic@vin.bg.ac.rs (J.M.); milica.nem@vin.bg.ac.rs (M.N.); 3 Laboratory of Bioactives, Health and Environment, Department of Biology, Faculty of Sciences, Moulay Ismail University of Meknes, Meknes 50050, Morocco; l.elrhaffari@umi.ac.ma

**Keywords:** in vitro, antibacterial activity, antibiofilm activity, crystal violet assay, essential oils, microdilution, root canal infection, root canal irrigant, *Enterococcus faecalis*

## Abstract

Background: Essential oils have gained in significance due to their various biological activities, and there is a growing demand for them in many industries. The present article focuses on the technical steps for an in vitro evaluation of the antibacterial and antibiofilm activities of essential oils for potential use as root canal irrigant in dentistry. Methods: The bioactivities of the essential oil were investigated through in vitro assays. The gram-positive bacterium *Enterococcus faecalis* was used as a micro-organism model. The antibacterial activity of the essential oil was assessed using the microdilution method, and resazurin staining to determine the minimal inhibition concentrations (MICs) and the minimal bactericidal concentrations (MBCs). The antibiofilm effect was evaluated spectrophotometrically at 570 nm using the microplate cultivation technique and crystal violet staining. Conclusions: This article features a detailed in vitro protocol to facilitate the preparation of the essential oil samples, the bacterial suspension, and the methods used for assessment of the antibiofilm and antibacterial activities of the essential oil. The advantages of these approaches are presented in relation to the limits linked to the choice of the bacteria and the essential oil.

## 1. Introduction

Infections, particularly recurrent ones, have been widely described in dentistry. These infections are often caused by bacteria that form resilient biofilms, which present a significant challenge for effective treatments [1]. The composition of biofilms in dentistry may vary considerably depending on the infection type and geography, but chronic and recurrent infections are generally caused by bacteria that are resistant to immune system defense and antimicrobials [2]. The endodontic infection is one of the most tenacious forms of infection, that is caused by resilient microorganisms either sheltered within biofilms and/or concealed in the complexities of the root canal system. Root canal infection often results from untreated dental caries, leading the infection to gradually migrate in an apical direction, until it reaches the periapical tissues and causes inflammation called “apical periodontitis” [1]. The global occurrence of apical periodontitis and the frequency of root-filled teeth is significant, with approximately 55% of the world’s population possessing at least one root-filled tooth, and 52% of the population having at least one instance of apical periodontitis [3]. For many years, the standard approach to successful endodontic treatment has evolved around (i) shaping using special files to remove remaining inflamed and/or infected pulpal tissue, (ii) cleaning using root canal irrigants, and (iii) filling of the root canal system. Among these three key steps of root canal therapy, irrigation is the cornerstone of the healing of periapical tissues [4]. Despite the huge advancement in the science of endodontics, especially in the performance of shaping files, the importance and the dedicated time frame for irrigation have remained constant, since the untreated areas after instrumentation can reach 50% of the total surface [5]. Consequently, this difficulty emphasizes the importance of using efficient antimicrobials as irrigating solutions during the disinfection [1].

Sodium hypochlorite (NaOCl) is currently considered the gold standard irrigant solution, because of its remarkable ability to eradicate biofilms. NaOCl is commonly used in concentrations ranging from 1.5% to 5.25% to effectively dissolve both organic tissue (pulp residue) and microbial biofilms [6]. However, NaOCl presents several disadvantages, mainly toxicity by causing tissue irritation, especially in the case of immature teeth with larger apices, where maintaining apical integrity and stem cells from the apical papilla is crucial for pulp regeneration therapies [7]. Additionally, NaOCl has the potential to cause allergic reactions for certain patients and practitioners. Also, it was noticed that NaOCl can lead to the fragilization of the root structure by compromising the biomechanical properties of dentine (collagen degradation and deproteination) especially when used in high concentrations and long periods, leading to a significant reduction in resistance and microhardness [8]. Another commonly used irrigant in endodontics is chlorhexidine (CHX), which is clinically used at concentrations of 0.2% to 2%. CHX has been proposed as an alternative for its antimicrobial activity and lower toxicity when compared to NaOCl [6]. However, CHX does not dissolve organic tissues or eradicate biofilms like NaOCl does [9].

To bypass these problems, disinfecting agents and irrigation protocols are constantly being tested. Amongst other approaches, natural agents have been proposed as novel adjuvant antimicrobial agents. Essential oils (EOs) are one of these natural antimicrobial products that have emerged as promising agents because of their potential to be integrated into formulations of auxiliary agents in endodontics. EOs have demonstrated efficacy against a wide range of bacteria, including both Gram-positive and Gram-negative species [10]. They are known for their multitarget action owing to their multicomponent nature, which makes it difficult for bacteria to develop resistance. Additionally, the terpenes and phenylpropanoids commonly present in these oils can interfere with various processes supporting biofilm resistance, such as adhesion [11], quorum sensing [12], and modulation of metabolic pathways [13]. However, before exploring these processes, it is important to subject these agents to preliminary antibacterial and antibiofilm screening, using simple in vitro tests. The assessment of antibacterial activity is essential as it gauges an agent’s ability to inhibit or eradicate bacteria in their planktonic form. Therefore, to determine the effectiveness of an antibacterial agent, the minimum inhibitory concentration (MIC) and the minimum bactericidal concentration (MBC) should be considered. The MIC represents the lowest concentration of an antimicrobial that inhibits the visible in vitro growth of microorganisms. The MBC represents the lowest concentration of an agent required to completely eliminate bacteria. In this article, the liquid microdilution method in a polystyrene 96-well microtiter plate was proposed for determining the MICs and MBCs. The assessment of the antibiofilm potential of an antimicrobial agent is also essential, as it determines its capacity to disrupt or prevent the formation of bacterial biofilms. This activity can be assessed using various tests, such as crystal violet assay, XTT assay, live/dead staining, and confocal laser scanning microscopy. In this article, the crystal violet assay was proposed.

The primary aim of this methodological study is to provide a detailed description and visualization of two chemical methods, microdilution assay and crystal violet assay, utilized to assess the antibacterial and the antibiofilm potential of EOs. 

## 2. Experimental Design

**Figure 1:**
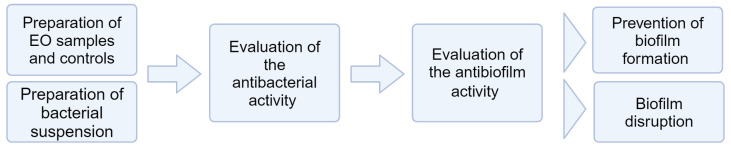
Methodological flowchart.

The protocol outlined describes the steps for the assessment of the antibacterial and antibiofilm potential of an EO for its use as a root canal irrigant (Figure 1). The details of each step are provided in clear and high-resolution images to serve as a visual aid in the subsequent figures. *Enterococcus faecalis* (*E. faecalis*) was proposed as a model bacterium to illustrate the steps of antibacterial and antibiofilm assays. In fact, *E. faecalis* is usually observed in teeth with persistent root canal infections. Its resilience and adaptability to conditions within the root canal make it extremely difficult to eradicate using conventional root canal treatment [14].

### 2.1. Materials

1. Rezasurin Dye (Thermo Scientific, Fair Lawn, NJ, USA; Cat. no.: 418900250);
2. Crystal violet Dye (CV; Bio-Merieux, Marcy-l’Étoile, France);
3. Blood agar (Tryptone peptone, Yeast extract, Sodium chloride, sheep blood, agar; Torlak, Belgrade, Serbia);
4. Mueller–Hinton agar (MHA) (HiMedia, Thane, India);
5. Tri-antibiotic paste (TAP) (metronidazole (Orvagyl^®^; Galenika, Belgrade, Serbia; 400 mg), minocycline (Minocin^®^; Pfizer, New York, NY, USA; 100 mg), and ciprofloxacin (Ciprofloxacin^®^; Remedica LTD, Limassol, Cyprus; 200 mg);
6. Dimethyl sulfoxide (DMSO; VWR Prolabo, Paris, France);
7. Polyoxyethylene sorbitan monooleate (Tween 80; Fisher Scientific, Geel, Belgium);
8. Tryptic soy broth (TSB; Torlak, Belgrade, Serbia);
9. Ethanol 96% (Zorka Pharma Hemija, Šabac, Serbia);
10. Saline (Hemofarm, Vršac, Serbia; Cat. no.: 13TD7A).

### 2.2. Equipment

1. Pipette tips (HDMED, Yancheng, Jiangsu, China; Cat. no.: BN3012);
2. Petri plates (LLG Labware, Hamburg, Germany);
3. Eppendorf tubes (Eppendorf, Hamburg, Germany);
4. Incubator (Orbital Shaker-Incubator ES-20; Grant-bio, Cambridge, England);
5. Flat bottom 96-well microplate (96-Well Microtest Plates; Sarstedt, Germany);
6. Micropipettes (Dragonlab, Beijing, China);
7. Absorbance reader (Micro-plate reading spectrophotometer; Thermo, Scientific Multiscan FC, Vantaa, Finland);
8. Spectrophotometer (UV-6300 PC Double Beam Spectrophotometer; MRC Scientific instruments, Holon, Israel);
9. Laminar (BIO-CL-130; EHRET Gmbh & CO.KG, Emmendingen, Germany).

## 3. Procedure

### Preliminary Preparations

The EO samples, positive control, and bacterial suspensions were prepared as follows.

**Figure 2:**
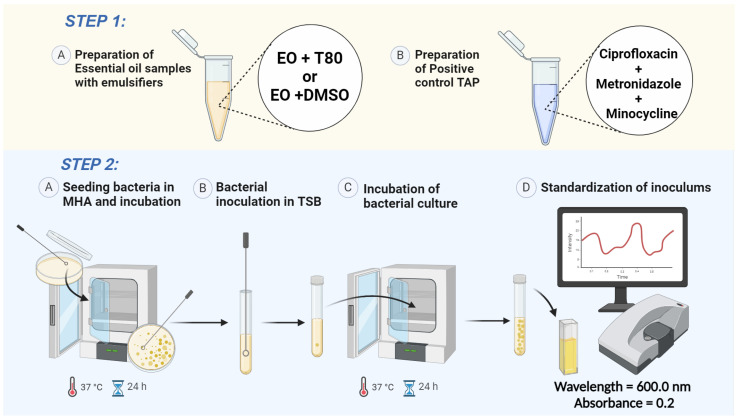
Preparation of essential oil samples, positive control, and bacterial suspensions. EO: essential oil; T80: Tween 80; DMSO: dimethyl sulfoxide; TAP: tri-antibiotic paste; MHA: Mueller–Hinton Agar; TSB: tryptic soy broth. Created with BioRender.com.

*Step 1*: Preparation of EO samples and positive control (Figure 2, Step 1).

EO was dissolved independently in two emulsifiers, dimethyl sulfoxide (DMSO) and polyoxyethylene sorbitan monooleate (Tween 80), at a ratio of 1:1, i.e., 10 µL of EO was added to 10 µL of either DMSO or Tween 80. The final volume of each test samples was 20 µL. A tri-antibiotic paste (TAP) was used as the positive control. Considering that TAP is composed of a mixture of ciprofloxacin, metronidazole, and minocycline, it is not only used as a root canal medication but is also suitable for 24 h testing because of its proven extended action. NaOCl cannot be used as a positive control for the microdilution method due to its short-term effect [15]. 

*Step 2*: Preparation of bacterial suspensions (Figure 2, Step 2).

Frozen clinical isolates of *E. faecalis* [16] were activated by streaking frozen stock cultures on Mueller–Hinton agar (MHA) media (composed of 3% (*w*/*v*) HM infusion, 1.75% (*w*/*v*) acicase, 1.7% (*w*/*v*) agar, and 0.15% (*w*/*v*) starch with the pH adjusted to 7.2–7.4) and incubated for 24 h at 37 °C. From the MHA plates, the bacterial colony was seeded into tryptic soy broth (TSB) media (containing 1.7% (*w*/*v*) tryptone, 0.3% (*w*/*v*) soya peptone, 0.5% (*w*/*v*) sodium chloride, and 0.25% (*w*/*v*) dextrose with the pH adjusted to 7.1–7.5) and incubated overnight at 37 °C. To ensure an identical initial bacterial concentration for each experiment, the inoculums were adjusted spectrophotometrically to an OD600 (absorbance at 600 nm) of 0.2, corresponding to 1 × 10^8^ CFU/mL. 

**Figure 3:**
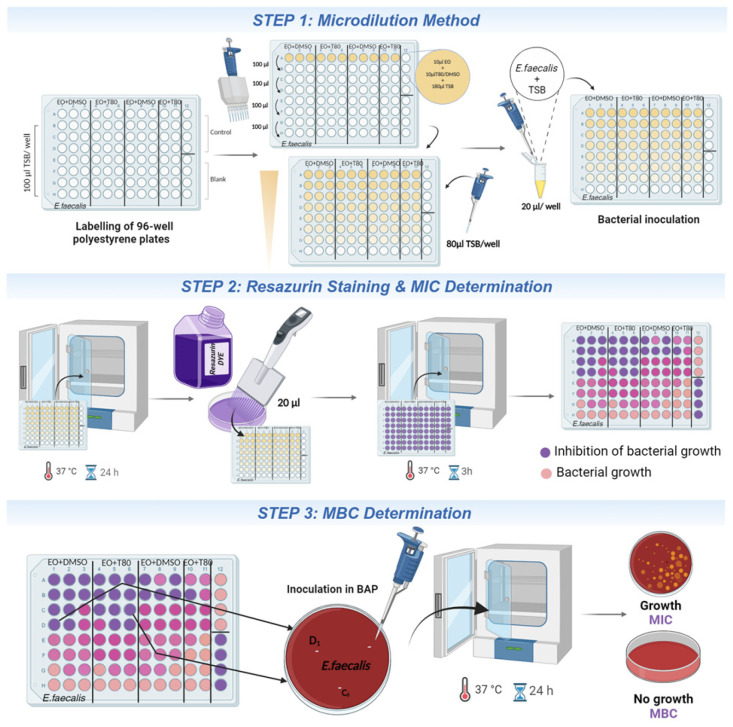
Procedure 1: EO’s antibacterial activity assessment determining MIC and MBC values. EO: essential oil; T80: Tween 80; DMSO: dimethyl sulfoxide; MIC: minimum inhibitory concentration; MBC: minimum bactericidal concentration; TSB: tryptic soy broth; BAP: blood agar plate. Created with BioRender.com.

**Procedure 1:** Determination of the MIC and MBC values for antibacterial activity assessment (Figure 3).

The antibacterial activity of the EOs was investigated to determine their effects on planktonic bacterial cells using the microdilution method and to determine the minimum inhibitory concentration (MIC) and bactericidal concentration (MBC). 

*Step 1:* The microdilution method was used to assess the impact of EO on bacteria over a 24 h period. A sterile flat-bottomed polystyrene 96-well microtiter plate was used for the microdilution assay. Each plate was carefully labeled with the name of the bacterial species under investigation, along with the name of the EO and its emulsifier. The positive control and blank (negative control) wells were also designated. The tested EO concentrations were obtained by a two-fold dilution. Initially, EO samples (20 µL) were diluted in 180 µL of sterile TSB medium (first row of the plate). Then, a serial dilution of the samples was proposed as follows: 100 µL of the well content (from the first row) was drawn and transferred to wells containing a volume of 100 µL of TSB using a multichannel micropipette. This process was repeated for each row, with 100 µL discarded from the last row, resulting in all the remaining wells containing a total volume of 100 µL. After serial dilution, an additional TSB (80 µL) was added to each well. Finally, each well was inoculated with 20 µL of the standardized bacterial suspension at a concentration of 1 × 10^6^ CFU/mL (Figure 3, Step 1).

*Step 2:* After 24 h of incubation of the microtiter plates at 37 °C, the resazurin dye was added to the wells (20 µL) and the microtiter plates were further incubated under dark conditions at 37 °C for 3 h to allow resazurin reduction to take place. The wells with no resazurin color change corresponded to the MIC values representing the lowest concentrations of EO at which there was no visible bacterial growth (Figure 3, Step 2).

*Step 3:* To determine the MBC, 15 μL from the wells corresponding to the concentrations equal and higher than the MIC were seeded onto blood agar plates (BAP). Then, the plates were incubated for 24 h at 37 °C. The MBC value corresponded to the lowest concentrations of EO in which no bacterial growth was detectable on the plates. All manipulations were performed under aseptic conditions in a laminar flow hood (Figure 3, Step 3).

**Figure 4:**
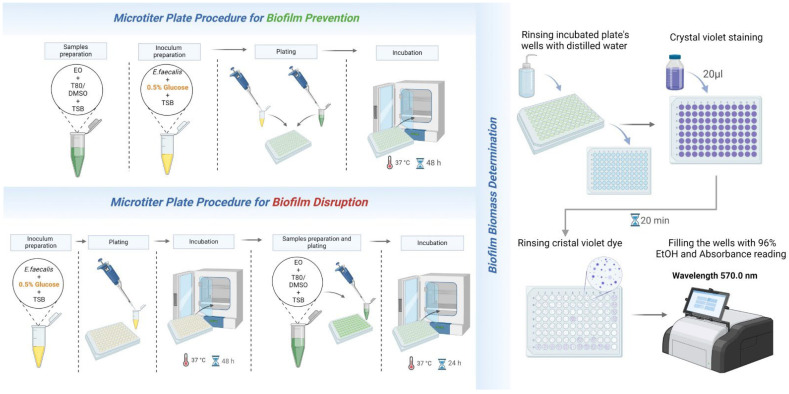
Procedure 2: EO’s antibiofilm activity assessment. EO: essential oil; T80: Tween 80; DMSO: dimethyl sulfoxide; TSB: tryptic soy broth. Created with BioRender.com.

**Procedure 2:** Assessment of the antibiofilm activity (Figure 4).

The assessment of the antibiofilm activity of the EO involved evaluating its effect on biofilm formation and biofilm disruption. EO samples were usually tested at MIC/8, MIC/4, MIC/2, and MIC concentrations when evaluating their potential for biofilm formation. However, when assessing their ability to disrupt pre-existing biofilms, MIC/2, MIC, 2MIC, and 4MIC were the usual concentrations used. Wells with only the bacterial strain and medium were considered as the negative control. This assay was conducted in polystyrene 96-well microtiter plates, and crystal violet staining was used to estimate the effect of EO on total biofilm biomass. 

*For the biofilm prevention assay*, EO samples were added to the wells at the previously mentioned concentrations, and bacterial inoculums with TSB media enriched with 1% glucose (to stimulate bacterial biofilm growth) were added simultaneously. The plates were then incubated for 48 h at 37 °C. 

**Figure 5:**
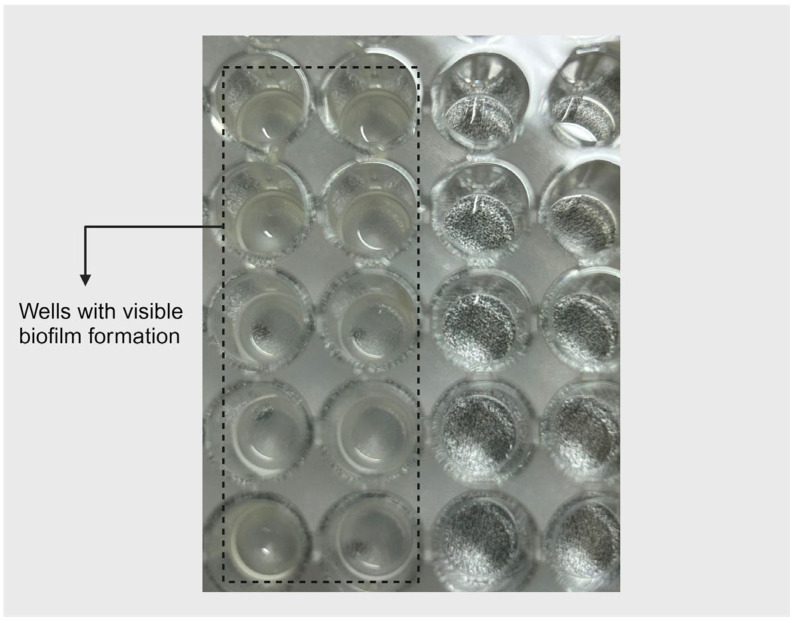
Bacterial biofilm development in a polystyrene 96-well plate.

*For the biofilm disruption assay*, *E. faecalis* biofilm was grown in a microtiter plate where TSB medium (enriched with 1% glucose) was used as a growth medium for 48 h at 37 °C. Bacterial growth was compared to untreated-control wells (cell growth) and medium-control wells (no cell growth) (Figure 5). After biofilm formation, the medium was aspirated using a multichannel pipette. EO samples at the aforementioned concentrations were then added to the pre-formed biofilm. 

*Biofilm biomass quantification*: After incubation, the supernatant was discarded from each well using a multichannel pipette, and the wells were rinsed twice with distilled water and air dried. After drying, each well was stained with crystal violet dye (200 µL) and carefully rinsed after 20 min. To resuspend the dye and enable reading of the results, 200 µL of 96% ethanol was added to each well. Optical density was read at a wavelength of 570 nm on a spectrophotometer, and the inhibition of biofilm formation and biofilm disruption by the tested substances was assessed by comparing the absorbance values obtained from treated wells and untreated control wells. A decrease in absorbance indicated a reduction in the biofilm biomass, suggesting antibiofilm activity. Three independent experiments, with five replicates, were conducted for each preparation. The percentage inhibition (%) of biofilm formation was calculated using the following formula:

$$I\left(\%\right)=\frac{\left(AC-AT\right)}{AC}\times 100$$

where *AC* and *AT* represent the absorbance at 570 nm of the control and treated groups, respectively.

## 4. Expected Results and Discussion

EOs are volatile liquid fractions commonly derived from plant materials such as stems, leaves, roots, wood, and resins [17]. The efficacy of EOs in combating bacteria and biofilms is strictly connected to their chemical composition, which is influenced by numerous factors such as the provenance of the plant, the part of the plant used, the stage of plant development, climatic and growth condition (temperature, soil, fertilizers, etc.), as well as storage conditions and the extraction techniques. EOs can be extracted using a variety of methods, which can be categorized into traditional and advanced techniques. While traditional methods like hydrodistillation and steam distillation are widely used, recent studies have suggested that innovative microwave-assisted methods (microwave hydrodiffusion and solvent-free microwave extraction) and ultrasound-assisted extraction techniques are more efficient and offer several benefits. Elyemni et al. showed that a microwave hydrodiffusion technique reduced the extraction time, energy utilized, and solvent consumption compared to a conventional hydrodistillation [18]. Additionally, these advanced methods have been shown to also improve the yield and the quality of the extracted oils [17]. EOs are widely used across industries, including food, medical, pharmaceutical, and cosmetics [19]. Given the growing demand for these natural products, it is crucial to explore their potential for antibacterial and antibiofilm activity. 

The antibacterial and antibiofilm effects of various EOs were assessed and validated using the microdilution and crystal violet assays in several studies. Marinkovic et al. [16] tested two EOs *Cymbopogon martinii* and *Thymus zygis* for their antibacterial and antibiofilm activities. For the antibacterial assessment, a microdilution assay was used to obtain the MIC and MBC against *Streptococcus mitis*, *Streptococcus sanguinis*, and *E. faecalis* planktonic cells. For the antibiofilm assessment, the crystal violet assay was used to evaluate the antibiofilm efficacy of *C. martinii* and *T. zygis* on single species biofilms and a multispecies biofilm composed of *S. mitis, S. sanguinis*, and *E. faecalis* in vitro using microtiter plates. For the ex vivo assay, the EOs were tested within the root canals of extracted premolars using scanning electron microscopy. For the multispecies biofilm formed in microtiter plates, *C. martinii* showed a biofilm reduction at a concentration of 4×MIC. For the multispecies biofilm formed in root canals, two different protocols were tested as follows: (i) the evaluation protocol, where *C. martinii* and *T. zygis* EOs were tested individually for their antibiofilm efficacy and were compared to 1.5% NaOCl and (ii) the supporting protocol, where the EOs were used as final irrigants after initial irrigation with 1.5% NaOCl. For the evaluation protocol, *C. martinii* showed similar effectiveness as the control group treated with 1.5% NaOCl. However, *T. zygis* showed higher effectiveness at inhibiting bacterial growth compared to both *C. martinii* and 1.5% NaOCl. For the supporting protocol, the efficacy of the final irrigation with *C. martinii* proved to be more efficient in reducing biofilms compared to the control group (1.5% NaOCl alone) as it enhanced the antibiofilm efficacy of the treatment. Similar results were observed with *Cymbopogon citratus* EO when tested against *E. faecalis* biofilm. The results of the crystal violet assay showed that *C. citratus* EO inhibited biofilm formation while the cell viability assay showed that it exhibited a high intracanal biofilm disruption when used as a final irrigation solution compared with the negative control (without EO) and with the sole NaOCl [20]. 

Apart from their potential application in endodontics, EOs have been also studied for their antimicrobial properties for potential use in preventing dental caries and treating periodontal diseases. In fact, various Eos, such as *Croton cajucara* and *Ocimum americanum*, have demonstrated significant antimicrobial activity and comparable efficacy to CHX against the cariogenic bacteria *Streptococcus mutans* and *Lactobacillus lactis*. This activity was attributed to the presence of certain compounds in these oils, such as linalool, which effectively inhibit bacterial growth and biofilm formation [21]. Other EOs, like *Lippia sidoides*, have shown clinical efficacy against periodontal pathogens, exhibiting significant decreases in gingival bleeding and subgingival plaque similar to 0.12% CHX after 7 day trial [22].

Evaluating antibiofilm activity is essential for comprehending and combatting biofilm-related infections. In this article, the crystal violet assay was employed for quantifying the biofilm biomass. However, it is crucial to opt for the appropriate method based on the specific objectives of the study and the characteristics of the EO being tested, as each method possesses its own strengths and limitations. For instance, while the XTT assay measures the metabolic activity of biofilms, it provides additional insights into the viability of biofilm cells rather than just biomass and can be considered as a viable alternative to the crystal violet assay [23]. Moreover, while culturing methods such as the disc dilution and tube dilution assay are capable of evaluating the efficacy of an EO against a specific species of bacteria or biofilm, molecular methods such as polymerase chain reaction can also offer insights into the underlying mechanisms of action of these EOs [24]. Microscopy techniques such as confocal laser scanning microscopy, scanning electron microscopy, and fluorescence microscopy, on the other hand, can allow for the visualization of the interactions between the EO and the bacteria showing the impact on bacterial viability and biofilm disorganization [25]. Overall, a combination of these methods can provide a comprehensive understanding of the antibiofilm properties of EOs, which can be useful in the development of novel therapeutic agents. Concerning the comparison group used to assess the antibacterial activity of the EO samples, TAP was chosen in our study in order to provide a known standard in endodontics that is used for antibacterial efficacy for comparison, knowing that it is primarily used as an intracanal medication. However, other solutions can be used as the control at different concentration such as CHX [26] and MTAD (a mixture of tetracycline, acid, and detergent) [27]. 

In this study, the antibiofilm activity of EO was tested on a 48 h single-species biofilm. However, since the biofilm was relatively young, the results may have overestimated the antibiofilm effectiveness of the EO. It is recommended to use mature and multispecies biofilms when assessing the antibiofilm efficacy of EOs in vitro, as these better represent the condition of root canal infections during treatment [28]. In order to ensure a biofilm’s maturity, it is important to remove non-adhered bacteria and metabolic by-products regularly. This can be achieved by refreshing the media on a weekly basis, to maintain optimal growth conditions for the biofilm [4]. Therefore, researchers can achieve more robust and reliable results in their experiments.

In this study, 96-well polystyrene microtiter plates were utilized as a substrate for cultivating *E. faecalis* biofilm in order to evaluate the in vitro effect of the EO. The use of synthetic substrates, such as polystyrene microtiter plates, allows for better standardization. However, these synthetic substrates may impact the initial stages of biofilm formation, as bacteria adhere better to dentinal collagen, which may influence the biofilm structure and composition [29]. Furthermore, synthetic substrates are unable to replicate the unique structure and properties of dentine which can affect the chemical interactions between EOs and microorganisms. Therefore, natural surfaces, such as human or bovine dentine, can be utilized to investigate the antibiofilm effect of EOs in order to provide insight into the potential clinical applications of these oils [30].

## 5. Limitations

Various limitations could be considered when evaluating the antibacterial and antibiofilm activities of EOs. First, the use of clinical strains to evaluate the antibacterial and antibiofilm effectiveness of an EO could influence the results due to the difference in the sensitivity and virulence of the clinical strain. Thus, it is important to include more than one clinical strain of the same bacterial species. Reference strains could also be important to study, as they provide a standardized and controlled environment for evaluation. In addition, they make it possible to compare results across different studies and laboratories [31]. However, it is important to mention that some reference strains have a limited ability to form biofilms, which makes it difficult to assess antibiofilm activity [32]. Second, some EOs have been shown to exhibit high efficacy against planktonic cells rather than biofilms, whereas other EOs have shown higher activity against biofilms. In fact, various studies have shown that Eos, such as sage and spearmint, *Massoia aromatica* and *Cinnamomum burmannii*, inhibited both the planktonic growth and biofilm formation of *Staphylococcus aureus*; however, higher concentrations were needed for biofilm disruption [33,34]. On the other hand, wild *Thymus vulgaris* EO showed a specific biofilm-related response as the MIC required to inhibit biofilm formation was much lower than that required to inhibit planktonic growth [35]. This variation in antibacterial and antibiofilm activities highlights the complexity of EO effects on different microbial aspects and suggests the necessity of evaluating both activities. Third, the microdilution method was optimized using Tween 80 to emulsify the EO. The usage of such emulsifiers has been shown to provide accurate and reliable results for testing the antimicrobial activity of EOs [36]. However, recent studies have highlighted some challenges related to the use of the pure forms of EOs, such as low water solubility, high volatility, and limited stability [37]. In order to overcome these limitations, da Silva Bruno Dutra et al. suggested nanotechnology-based delivery systems, in which EOs can be encapsulated into nanosized droplets called nanoemulsions. These EO nanoemulsions show improved antibacterial activity and long-term efficacy compared to their conventional forms because of their high stability, better dispersion, and increased solubility [37]. Several studies have demonstrated that EOs nanoemulsions exhibit higher antibacterial effects against pathogens compared to their non-nano counterparts. Marinkovic et al. investigated *C. citratus* EO nanoemulsion for its antibacterial and antibiofilm properties to be used as an auxiliary root canal disinfectant. The EO-based nanoemulsion was tested within the root canals of extracted teeth against *E. faecalis* biofilm. The results provided by the screening of the antibiofilm effect showed the remarkable potential of the EO in the form of nanoemulsion irrigant compared with that of the sole EO [20]. Despite the limitations mentioned above, this article thoroughly described the key steps involved in evaluating the potential of EOs to be used as root canal disinfectants. It also presented a thorough overview of the two colorimetric methods, the crystal violet assay and the microdilution assay, as well as the preparation of EO samples and bacterial inoculums. Hence, this article, which features a detailed experimental design, serves as a valuable resource for researchers who wish to conduct antibacterial and antibiofilm activity studies on EOs.

## Data Availability

No new data were created or analyzed in the study.
